# Treatment of Periodontitis Affecting Human Primary Teeth—A Systematic Review

**DOI:** 10.3390/dj11070171

**Published:** 2023-07-14

**Authors:** Protyusha Guha Biswas, Anusha Mohan, Eswar Kandaswamy

**Affiliations:** 1 Department of Oral Pathology and Microbiology, Meenakshi Ammal Dental College and Hospital, Chennai 600095, India; drprotyusha.oralpathology@madch.edu.in; 2 AR Medical Center, Chennai 600116, India; anushamohan891@gmail.com; 3 Department of Periodontics, Louisiana State University Health Sciences Center, School of Dentistry, New Orleans, LA 70119, USA

**Keywords:** aggressive periodontitis, systematic review, human primary teeth, scaling and root planing, clinical attachment loss, probing depth

## Abstract

The aim of this systematic review is to report the treatment options (Intervention) and outcomes (O) for primary teeth affected by periodontitis (Population) and if the treatment of primary teeth can prevent the spread of periodontitis to permanent teeth (Outcomes). The following databases were searched for papers published before December 2022: PubMed, Embase, Web of Science, and Ebscohost. Studies on children affected by periodontitis involving the primary teeth were included and those on children who presented with periodontitis as a manifestation of systemic disease were excluded. Narrative synthesis and methodological quality assessments were performed for the included studies. Three interventional studies (without a control group) that evaluated treatments involving scaling and root planing (SRP with antibiotics) and extraction were included (total *n* = 60 patients). Additionally, twelve case reports/case series articles (*n* = 19 patients) were identified. The diagnoses ranged from aggressive periodontitis to juvenile periodontitis and pre-pubertal periodontitis. Based on a limited number of published studies, it was found that the early treatment of periodontitis affecting the primary teeth using SRP and systemic antibiotics resulted in favorable improvements in PD and CAL. Limited evidence suggests that SRP and the extraction of the primary teeth involved have the potential to prevent periodontitis affecting permanent teeth. Future trials are required to standardize the treatment protocols and to confirm these findings.

## 1. Introduction

Periodontitis is an inflammatory condition that is induced by microbial plaque and results in attachment loss and bone loss [1]. A form of periodontitis called Grade C molar-incisor periodontitis (formerly known as aggressive periodontitis) is a rapidly progressing form of periodontitis that is characterized by early onset and rapid attachment loss in a healthy individual [2]. In general, this form of periodontitis is reported to have a circum-pubertal onset and can affect permanent teeth. The involvement of primary teeth in this pattern of periodontitis can occur in systemically healthy children [3,4,5,6,7]. In addition, evidence has shown that patients presenting with Grade C molar-incisor pattern periodontitis affecting permanent dentition showed attachment loss that affected the primary teeth as well [3,8].

Before the release of the 2018 classification of periodontal and peri-implant diseases [9], periodontitis affecting the primary teeth was diagnosed using different terminologies, including juvenile periodontitis (localized and generalized), aggressive periodontitis (AP—both localized and generalized), rapidly progressive periodontitis, and pre-pubertal periodontitis [10]. However, the 2018 classification of periodontal and peri-implant diseases did not include a separate category for periodontitis affecting the primary teeth [9]. Treating patients with periodontitis that affects the primary teeth can be challenging, due to rapid disease progression and a lower periodontal attachment area compared to permanent teeth, which can lead to early tooth loss [11]. Tooth loss in growing patients has long-term consequences regarding the development of the jaws and the permanent teeth [12]. Since periodontitis can be treated predictably in its early stages, it is important to recognize this condition as soon as it manifests in the primary teeth [13]. The aim of treatment in such cases is to establish a healthy periodontium that can be maintained to minimize primary tooth loss and the spread of periodontitis to permanent teeth [11]. Currently, the available treatment modalities for periodontitis that affects the primary teeth include scaling and root planing (SRP) with or without adjunct antibiotics and the extraction of the affected teeth [5,6,8,14].

Several systematic reviews [15,16,17,18] have evaluated the various treatment modalities that are adopted for periodontitis that affects permanent dentition. However, to date, no systematic review has been published that reports the management of periodontitis affecting the primary teeth. Hence, the aim of this systematic review is to report the treatment options and outcomes for primary teeth that have been affected by periodontitis. The secondary aim of this review is to report if the treatment of primary teeth that have been affected by periodontitis can prevent the spread of periodontitis to permanent teeth.

## 2. Materials and Methods

The manuscript has been reported according to the PRISMA 2020 guidelines for reporting systematic reviews [19]. The review protocol has been published in PROSPERO ([20]) (Ref. No: CRD42019127469; version: January 2023).

### 2.1. Inclusion and Exclusion Criteria

Original research articles that reported the treatment outcomes of periodontitis affecting the primary teeth were considered for inclusion in the current review. Since the new 2018 classification for periodontal diseases does not include a separate category for the primary teeth [9], we used the term “periodontitis affecting primary teeth” to encompass previous definitions, including juvenile periodontitis (localized and generalized), AP (localized and generalized), rapidly progressive periodontitis, and pre-pubertal periodontitis involving the primary teeth. The population (P) studied comprised children with periodontitis affecting the primary teeth. The interventions (I) that were included were the use of SRP with or without antibiotics and extraction. The primary outcomes (O) of interest were an improvement in probing depth (PD) and clinical attachment loss (CAL), with a follow-up of at least 4–6 weeks. The secondary outcome (O) of interest was the appearance of periodontitis in permanent teeth after the treatment of primary teeth, recorded during the follow-up, as evaluated by clinical measurements with or without a radiographic evaluation. Studies performed on children with medical conditions (immune deficiencies or conditions that would be considered to fall under “periodontitis as a manifestation of systemic disease”) were excluded. The treatment of periodontitis that affected mixed dentition was included if separate data for the primary teeth were available. Case reports where no treatment was provided were excluded (Table 1).

### 2.2. Types of Included Studies

Initially, case-control, cohort studies, non-randomized studies of interventions, and clinical trials (with or without control groups) were included. However, an initial search revealed only three studies that met the inclusion criteria. The inclusion criteria were then broadened to encompass case series and case reports since several of these were identified during the search. Publications in languages other than English were excluded. Whenever missing information was encountered, the authors of that paper were contacted to elicit further information.

### 2.3. Search Strategy

The electronic search was performed in the following registries for papers published up to December 2022: PubMed, Embase, Web of Science, and Ebscohost. For the database searches, keywords and Boolean operators that were specific to each database were used to form the search strategy (the detailed search strategy is presented in the Supplementary Materials). The title and abstracts of all articles from the database searches were reviewed independently by two reviewers (EK, AM). The full texts were retrieved for all the included articles that were subsequently reviewed, to arrive at a final list of studies based on the criteria mentioned above. Any disagreements were noted and resolved in a discussion between the two reviewers and a third reviewer was available for consultation when necessary.

Apart from the database searches, an independent hand-search was performed in the European Journal of Paediatric Dentistry, Clinical Oral Investigations, and the European Archives of Paediatric Dentistry up to December 2022. Additional publications were searched by screening the bibliographies and reference lists from the included studies. Studies excluded after obtaining the full text were documented separately, along with the relevant reasons for exclusion (see the Supplementary Materials).

### 2.4. Data Extraction and Quality Assessment

Data were extracted from the included studies after the full-text screening. The data representing the primary outcomes (PD and CAL improvement) and secondary outcomes (permanent tooth involvement) were summarized using a narrative review and descriptive statistics.

The National Institutes of Health (NIH) tool [21], which is used for intervention studies with no control group, was utilized to assess the risk of bias in the included interventional studies. This tool was chosen since the interventional studies included in this paper had no control group. This tool evaluates the following criteria: objectives, eligibility criteria, representative population, sample size, intervention delivery, outcome measurement, follow-up, statistical methods, and multiple time point measurements [21]. An assessment of the risk of bias in the included studies was performed for the included case reports using the CARE [22] guidelines.

## 3. Results

### 3.1. Details of the Included Studies

**Figure 1:**
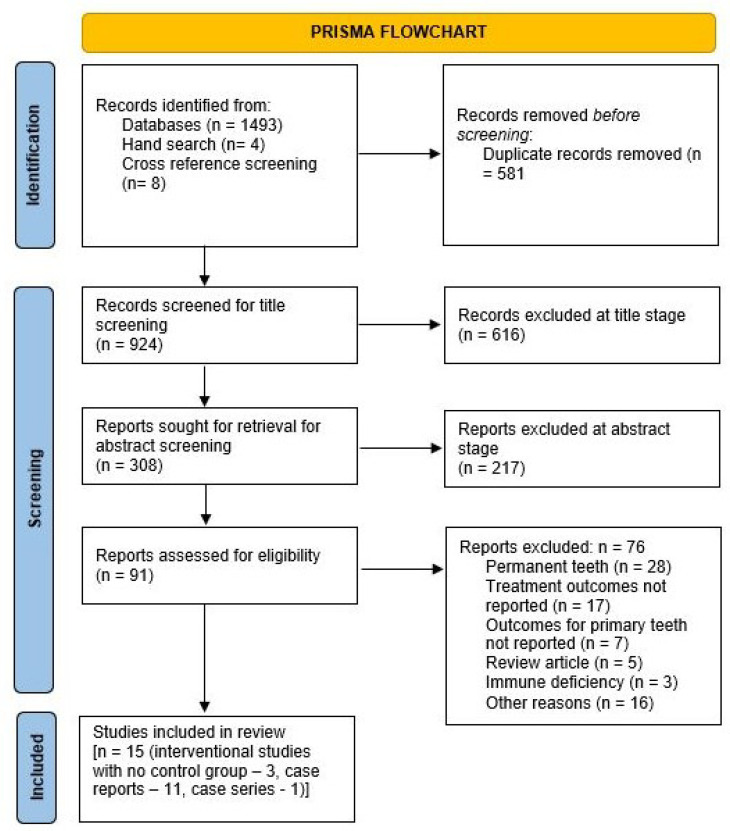
PRISMA flowchart for screening the articles.

The details of the search results are presented in the PRISMA flowchart shown in Figure 1. A total of 1505 articles were identified, with 1493 selected from the databases (PubMed: 596; Ebsco: 112; Embase: 438; Web of Science: 347). The treatment results for the patient population in one interventional study [23] were included in a subsequent follow-up paper [4] examining more patients and with a longer follow-up. However, the secondary aim of assessing the involvement of permanent teeth was only addressed in the former paper [23]; hence, both papers were included [4,23] for narrative synthesis. Additionally, two [24,25] case series reported the same case using different follow-up periods; hence, the paper with the longer follow-up [24] was included. In total, 11 case reports, 1 case series [5,6,7,14,24,26,27,28,29,30,31,32], and 3 interventional studies with no control group [4,23,33] were identified and included in the review (Figure 1). The characteristics of the included studies are described in Table 2 (interventional studies with no control group) including the number of cases, the treatment provided, follow-up, PD, and CAL at different time points, as well as the involvement of permanent teeth. Table 3 contains information regarding the included case reports, including patient age/sex, diagnosis, treatment provided, PD reduction, duration of follow-up, and the involvement of permanent teeth. The kappa value at the full-text screening stage was 0.53 (moderate agreement). A meta-analysis could not be performed since there was heterogeneity in the treatment protocols used in the interventional studies.

### 3.2. Description of the Included Interventional Studies

A total of 3 interventional studies with no control group were included in the present systematic review (Table 2). Miller et al. (2017) [4] reported the treatment outcomes in 27 systemically healthy patients with localized aggressive periodontitis (LAP) affecting the primary teeth. The affected teeth were treated using SRP, with adjunct systemic antibiotics (500 mg amoxicillin and 250 mg metronidazole three times a day for 7 days). A significant reduction in PD and gain in CAL was seen following treatment. While comparing the treatment outcomes in the primary and permanent teeth, a greater improvement in CAL and PD at 12 and 24 months was seen in the primary teeth. Merchant et al. (2014) [23] reported the results for 22 out of the 27 subjects presented in the article by Miller et al. (2017) [4]. They followed similar treatment protocols (SRP + amoxicillin and metronidazole) and reported significant reductions in PD and CAL gain at 3, 6, and 12 months following the treatment. There was a significantly higher gain in CAL at 3, 6, and 12 months for the primary teeth when compared to the permanent teeth. Additionally, 13/22 subjects showed the normal eruption of permanent teeth, while the remaining patients had primary teeth with LAP at the end of the follow-up period.

The third interventional study included in this review was conducted by Mros et al. (2010) [33] and involved 11 children between the ages of 7 and 13 with LAP. All the affected primary molars were extracted. The subjects were recalled at 14 to 19 years after treatment for a clinical examination with PD and BOP measurements being recorded and a radiographic examination including bitewings of the permanent teeth. At the follow-up examination, four of the recorded cases (36.3%) exhibited the development of periodontitis in permanent teeth, and the rest were free of periodontitis (Table 2). These findings, therefore, suggest that treatment of the primary teeth has the potential to prevent the spread of periodontitis to the permanent teeth.

### 3.3. Description of the Included Case Reports/Case Series

A total of 19 cases have been reported in the 12 included case reports/case series (Table 3). Of the ten cases reported in the paper by Bimstein et al. (2003) [24], one case did not include information regarding periodontitis affecting the primary teeth and was thus excluded; another case did not report the primary or secondary outcomes and was thus excluded, whereas the remaining 8 cases were included.

The included cases described children who were between 3 and 9 years of age. The common symptoms were bleeding from the gums, the mobility of one or more teeth, and the loss of teeth. In a case reported by Seremidi et al. (2012) [30], the mean PD of 7.5 mm was reduced to 3.8 mm following treatment over 18 months. Hazan-Molina et al. (2012) [5] reported a case where the initial PD was greater than 7 mm, which was reduced to 3 mm following treatment. Spoerri et al. (2014) [7] reported a PD of less than 4 mm at follow-up, while Ngan et al. (1985) [32] reported a PD within the normal range and Suzuki et al. (2003) reported a PD of within 2.5 mm after treatment.

Of the included case reports/case series, no cases reported periodontitis affecting the permanent teeth after treatment of the primary teeth. For case reports that pursued extraction as the treatment of choice, space maintenance options were reported in four case reports; these included pedi-partials [29], vacuum-formed removable retainers [5], space maintainers [24], and complete dentures (allowing space for the erupting permanent teeth) [6].

### 3.4. Quality Assessment of the Included Studies

The quality of the three interventional studies [4,23,33] was assessed using the NIH risk of bias tool and the provided guidance. Mros et al. (2010) [33] achieved a score of 6/12, Merchant et al. (2014) [23] achieved a score of 10/12, and Miller et al. (2017) [4] achieved a score of 10/12. Overall, the three studies showed a fair to low risk of bias (Table 4).

The quality of the included case reports/case series was evaluated using the CARE guidelines. All case reports except one were considered to be of high quality (>20) and the exception [32] was found to be of medium quality (10–20), while no articles were found to be of low quality (<10) (Table 5).

## 4. Discussion

Treatment outcomes for permanent teeth that have been affected by Grade C molar-incisor pattern periodontitis (formerly known as AP) have a better prognosis with early diagnosis and treatment [11]. In mild cases of Grade C, molar-incisor pattern periodontitis can be managed with non-surgical therapy using systemic antibiotics, followed by periodontal maintenance at regular intervals [34,35,36]. In more advanced cases, surgical treatment for the residual deep pockets and bone defects, followed by periodontal maintenance at regular intervals, is often needed [11,37,38,39].

Studies have reported that periodontitis can occur in healthy children [24,40], which can be an early sign of the potential involvement of permanent dentition [3,41]. If the affected primary teeth are not treated at an early stage, this can lead to spontaneous exfoliation or the need to extract the teeth at an early age, due to rapid attachment loss and disease progression [29,42]. The early diagnosis and treatment of periodontitis (affecting both the primary and mixed dentition) can provide an opportunity to limit the damage and prevent disease progression to permanent dentition [23], thereby improving the quality of life [43].

The included studies in this systematic review predominantly utilized SRP and extraction as the most common treatment modality for primary teeth that have been affected by periodontitis. Although the two treatment modalities (extraction and SRP) widely vary in their outcomes, the principal goal for primary teeth affected by periodontitis is to prevent the involvement of permanent dentition. In the studies included in this systematic review, both treatment modalities showed similar outcomes in terms of the potential to limit the involvement of the developing permeant teeth [4,23,33]. When SRP was used as a treatment modality, the included studies reported significant improvement in the clinical parameters (PD and CAL), especially when combined with adjunct systemic antibiotics [4,23]. The data available from the included case reports/series [5,7,30] in the systematic reviews reported similar findings to the included interventional studies. However, the limited sample size outlines the need for studies that are longitudinal in nature to confirm the findings and make evidence-based clinical recommendations.

The results of the included interventional studies in this systematic review suggest that the primary teeth responded better to SRP and adjunct antibiotics than the permanent teeth [4]. Although a direct comparison cannot be made, a reduction in PD after the SRP of permanent teeth is by about 1.3–2.1 mm, and the improvement in CAL is by about 0.6–1.2 mm [44]. When adjunct systemic antibiotics are prescribed along with SRP for permanent teeth, there is an additional 0.3–0.4 mm in terms of PD reduction and 0.15–0.2 mm in terms of CAL gain [45]. In contrast, the two included studies reported a 2.5-millimeter reduction in PD and a 3-millimeter improvement in CAL after SRP and systemic antibiotics were employed for the treatment of primary teeth with periodontitis [4,23], suggesting that early intervention in cases involving the primary teeth has better outcomes than in the case of permanent teeth, but future trials are required to confirm this observation.

This systematic review is not without its limitations. In the protocol for this systematic review, we included both interventional studies and case reports. Although case reports provide a low level of evidence, in the absence of randomized trials and limited observational studies, the data provided by case reports can be beneficial [46,47]. In the interventional studies included in the review, there was the absence of a control group and the relative sample sizes were low. Additionally, due to a lack of specific diagnostic terminology (according to the new 2018 classification of periodontal diseases) for periodontitis affecting the primary teeth, we have used the generic term “periodontitis affecting primary teeth” [9]. The search screening process aimed to identify only those articles written in the English language. The CARE guidelines were used to evaluate the risk of bias for the included case reports/series. Despite this being a reporting checklist, the CARE guidelines have been used previously for assessing the risk of bias in systematic reviews that have included case reports/series [46,47].

The systematic review identified the issue that there is a lack of standardized treatment protocols for periodontitis affecting the primary teeth, unlike the permanent teeth. SRP and extraction were most commonly employed in the included studies. The majority of the studies that employed extraction of the primary teeth as the treatment of choice did not report any information regarding space management. The management of concerns regarding space loss, the loss of esthetics, and the interruption of speech due to tooth loss need to be considered before choosing extraction as a treatment option [5,24,29]. Following treatment of the primary teeth, the results need to be evaluated by measuring PD and CAL and longitudinally reporting the effect on permanent dentition, as well as the effect on oral health-related quality of life. Future interventional trials are needed to determine the optimal treatment options for periodontitis affecting the primary teeth and to confirm if early intervention can prevent the involvement of permanent teeth.

## 5. Conclusions

Within the limitations of this systematic review, which is based on a limited number of studies, periodontitis affecting the primary teeth is currently managed by means of extraction or SRP (with or without antibiotic therapy). The included studies showed that treatment using SRP with antibiotic therapy results in a favorable reduction in PD and CAL. Additionally, the extraction and SRP of the affected primary teeth have the potential to prevent periodontitis from developing in the permanent teeth. Further interventional studies are required to standardize the treatment protocols.

## Figures and Tables

**Table 1:** Inclusion and exclusion criteria.

Inclusion	Exclusion
Subjects with periodontitis affecting the primary dentition. If mixed dentition was included, separate data for primary teeth were available.	Subjects with periodontitis as a manifestation of systemic disease.
Subjects receiving treatment for periodontitis affecting the primary teeth.	Subjects who received no treatment for periodontitis.
Subjects where the primary or secondary outcomes of interest were included.	Studies reporting only treatment results for permanent teeth.

**Table 2:** Data from the included interventional studies with no control group. Periodontal involvement of the permanent teeth was measured using clinical measurements (with or without radiographic data).

Author, Year, and Country	No. of Cases	Treatment Provided	Follow- Up	Probing Depth in mm Mean (SD)			Clinical Attachment Level in mm Mean (SD)			Permanent Tooth Involvement
				Initial	After Treatment		Initial	After Treatment		
					12 Months	24 Months		12 Months	24 Months	
Mros et al., 2010, Sweden [33]	11	Extraction	14 to 19 years		Not applicable			Not applicable		Yes (4 subjects had permanent tooth involvement)
Miller et al., 2017, USA [4]	27	SRP + amoxicillin and metronidazole	2 years	4.92 (0.7)	2.35 (1.15)	2.62 (0.51)	3.56 (1.42)	0.37 (0.58)	0.03 (0.10)	
Merchant et al., 2014, USA [23]	22	SRP + amoxicillin metronidazole	12 months	4.84 (0.65)	2.59 (1.15)	Not applicable	3.47 (1.44)	0.43 (0.64)	Not applicable	13/22 patients had no involvement of the permanent teeth; 9 patients had primary teeth involvement at the end of follow-up

**Table 3:** Data from the included case reports/case series.

Author, Year, and Country	Patient Age/Sex	Diagnosis	Treatment Provided	PD Reduction Following Treatment (mm)	Duration of Follow-Up	Permanent Tooth Involvement
Yoshida-Minami et al., 1995, Japan [26]	4 years/Male	Localized prepubertal periodontitis	Extraction and scaling with oxytetracycline chloride local administration (once a month for six months) The lower anterior teeth were irrigated with 0.02% benzalkonium chloride and antibiotic therapy (minocycline hydrochloride at 60 mg per day for 3 days) Local application of minocycline paste (once a day for one month)	Not reported	3 years	Unaffected until follow-up; measured using clinical and radiographic findings
Sixou et al., 1997, France [27]	5 years/Female	Prepubertal periodontitis	Extraction of the mobile teeth with antibiotic therapy (metronidazole for 3 weeks)	1–2 mm PD seen, no periodontal pockets	7years	Unaffected until follow-up; measured using clinical examination
Bimstein et al., 2003 [24]	9 years/Male	Prepubertal periodontitis	Extraction with antibiotic therapy (amoxicillin 375 mg and metronidazole 250 mg three times a day for 7 days)	Not reported	7 years	Unaffected until follow-up; measured using clinical and radiographic examination
	7 years/Male	Prepubertal periodontitis	Local and systemic antibiotics, with preventive therapy and monthly subgingival irrigation with chlorhexidine	Not reported	7 years	Unaffected until follow-up; measured using clinical and radiographic examination
	9 years/Female	Prepubertal periodontitis	Local and systemic antibiotic therapy	Not reported	7 years	Unaffected until follow-up; measured using clinical and radiographic examination
	9 years/Female	Prepubertal periodontitis	Local and systemic antibiotic therapy	Not reported	7 years	Unaffected until follow-up; measured using clinical and radiographic examination
	8 years/Female	Prepubertal periodontitis	Extraction No antibiotic therapy	Not reported	7 years	Unaffected until follow-up; measured using clinical and radiographic examination
	5 years/Male	Prepubertal periodontitis	Local and systemic antibiotic therapy was recommended; however, the patient did not receive the recommended antibiotic therapy	Not reported	7 years	Unaffected until follow-up; measured using clinical and radiographic examination
	3 years/Female	Prepubertal periodontitis	Extraction, local and systemic antibiotic therapy, and space maintainers	Not reported	7 years	Unaffected until follow-up; measured using clinical and radiographic examination
	5 years/Female	Prepubertal periodontitis	Extraction, antibiotic therapy (amoxicillin and metronidazole), and space maintainers	Not reported	7 years	Unaffected until follow-up; measured using clinical and radiographic examination
Suzuki et al., 2003, Japan [28]	5 years/Male	Localized aggressive periodontitis	Scaling, irrigation of pockets with iodine, extraction, cleaning, and irrigation (twice a month) and Minocycline paste (topical application)	Within 2.5 mm	2 years	Unaffected until follow-up; measured using clinical examination
Portaro et al., 2008, Spain [29]	3.7 years/Male	Generalized aggressive periodontitis	Extraction, scaling, and root planing with antibiotic therapy (amoxicillin 50 mg/kg/day and metronidazole 30/mg/kg for 10 days)	Not reported	4 years	Unaffected until follow-up; measured using clinical and radiographic examination
Hazan-Molina et al., 2011, Israel [5]	7 years/Female	Aggressive periodontitis	Scaling, root planing, and extraction with space maintainers in the form of vacuum-formed removable retainers	3 mm PD surrounding all teeth except primary maxillary left canine—10 mm	2 years (recall made every 3 months)	Unaffected until follow-up; measured using clinical and radiographic examination
Cunha et al., 2012, Brazil [6]	4 years/Female	Generalized aggressive periodontitis	Extraction and antibiotic prophylaxis for 48 h (250 mg amoxicillin 2 h before and at 6-hour intervals after surgery). A complete denture was given following the extraction	Not applicable	12 years	Unaffected until follow-up; measured using clinical examination
Seremidi et al., 2012, Greece [30]	8 years/Male	Generalized aggressive periodontitis	Extraction of the primary teeth with per-apical lesions. Scaling and root planing, antibiotic therapy (amoxicillin 50 mg/kg and metronidazole 30 mg/kg three times a day for 2 weeks) and 0.2% chlorohexidine mouth rinse for 10 days	3.8 (mean PD)	1.5 years (monthly recall for the first six months and every three months thereafter)	Unaffected until follow-up; measured using clinical and radiographic examination
Hilgers et al., 2004, USA [31]	6 years/Female	Localized aggressive periodontitis	Extraction with antibiotic therapy (Augmentin for 10 days (40 mg/kg/day, divided into 3 doses), later changed to metronidazole at 250 mg for 14 days, with a chlorhexidine rinse twice daily	<2 mm	1.5 years (recall with prophy every 3 months)	Unaffected until follow-up; measured using clinical and radiographic examination
Spoerri, et al., 2014, Switzerland [7]	4 years/Female	Generalized aggressive periodontitis	Extraction, scaling and root planing, antibiotic therapy (amoxicillin 350 mg three times a day and metronidazole 100 mg three times a day for 7 days), and 0.2% chlorhexidine application for 7 days	<4 mm	4 years (recall made every 6 months for first two years)	Unaffected until follow-up; measured using clinical and radiographic examination
Mass et al., 2018, Israel [14]	3 years/Male	Localized aggressive periodontitis	Local application of 0.2% chlorhexidine twice a day for 10 days. Scaling and curettage with antibiotic therapy (amoxicillin 250 mg three times a day for seven days)	<4 mm	2.2 years. At follow-up, 83 had exfoliated	Unaffected until follow-up; measured using clinical and radiographic examination
Ngan et al., 1985 [32]	8.5 years/Male	Advanced periodontitis	Extraction, oral prophylaxis and scaling every 6 months, tetracycline therapy (250 mg, 4 times a day, for 30 days), space maintainers	Within the normal range	2.5 years	Unaffected until follow-up; measured using clinical and radiographic examination

**Table 4:** Quality assessment of the included interventional studies, using the NIH risk of bias tool for those intervention studies with no control group. The criteria were derived from: https://www.nhlbi.nih.gov/health-topics/study-quality-assessment-tools (accessed 5 January 2023).

Criteria	Mros et al., 2010 [33]	Miller et al., 2017 [4]	Merchant et al., 2014 [23]
1. Was the study question or objective clearly stated?	Yes	Yes	Yes
2. Were eligibility/selection criteria for the study population prespecified and clearly described?	No	Yes	Yes
3. Were the participants in the study representative of those who would be eligible for the test/service/intervention in the general or clinical population of interest?	Yes	Yes	Yes
4. Were all eligible participants that met the prespecified entry criteria enrolled?	Yes	Yes	Yes
5. Was the sample size sufficiently large to provide confidence in the findings?	No	Yes	Yes
6. Was the test/service/intervention clearly described and delivered consistently across the study population?	Yes	Yes	Yes
7. Were the outcome measures prespecified, clearly defined, valid, reliable, and assessed consistently across all study participants?	Yes	Yes	Yes
8. Were the people assessing the outcomes blinded to the participants’ exposures/interventions?	Not reported	No	Not reported
9. Was the loss to follow-up after baseline 20% or less? Were those lost to follow-up accounted for in the analysis?	Yes	Yes	Yes
10. Did the statistical methods examine changes in outcome measures from before to after the intervention? Were statistical tests done that provided p values for the pre-to-post changes?	No	Yes	Yes
11. Were outcome measures of interest taken multiple times before the intervention and multiple times after the intervention (i.e., did they use an interrupted time-series design)?	No	Yes	Yes
12. If the intervention was conducted at a group level (e.g., a whole hospital, a community, etc.) did the statistical analysis take into account the use of individual-level data to determine effects at the group level?	Not applicable	No	No

**Table 5:** Quality assessment of the case reports/case series using the CARE guidelines.

Article	Year	Total Score (max 30)	Title (1)	Keywords (1)	Abstract (4)	Introduction (1)	Patient Information (4)	Clinical Findings (1)	Timeline (1)	Diagnostic Assessment (4)	Therapeutic Intervention (3)	Follow-up and Outcomes (4)	Discussion (4)	Patient perspective (1)	Informed Consent (1)
Yoshida-Minami et al. [26]	1995	22	0	0	4	1	3	1	1	2	2	4	4	0	0
Sixou et al. [27]	1997	21	0	0	4	1	3	1	1	2	2	3	4	0	0
Bimstein [24]	2003	22	0	0	4	1	4	1	1	2	2	3	4	0	0
Suzuki et al. [28]	2003	21	1	0	4	1	4	1	0	1	2	3	4	0	0
Portaro et al. [29]	2008	21	1	0	4	1	3	1	0	2	2	3	4	0	0
Hazan-Molina et al. [5]	2011	21	1	0	4	1	3	1	0	2	2	3	4	0	0
Cunha et al. [6]	2012	21	1	0	4	1	3	1	0	2	2	3	4	0	0
Seremidi et al. [30]	2012	21	0	0	4	1	3	1	0	2	2	4	4	0	0
Hilgers et al. [31]	2004	24	1	0	4	1	4	1	1	2	3	3	4	0	0
Spoerri et al. [7]	2014	20	0	0	4	1	3	1	0	2	2	3	4	0	0
Mass et al. [14]	2017	21	0	0	4	1	4	1	0	2	2	3	4	0	0
Ngan et al. [32]	1985	18	1	0	2	1	3	1	0	2	2	3	3	0	0

## Data Availability

The data used in this study is taken from previously published literature.

## Supplementary Materials

The following supporting information can be downloaded at: https://www.mdpi.com/article/10.3390/dj11070171/s1, Supplementary Material S1. References [48,49,50,51,52,53,54,55,56,57,58,59,60,61,62,63,64,65,66,67,68,69,70,71,72,73,74,75,76,77,78,79,80,81,82,83,84,85,86,87,88,89,90,91,92,93,94,95,96,97,98,99,100,101,102,103,104,105,106,107,108,109,110,111,112,113,114,115,116,117,118] are cited in the Supplementary Materials.
