# Supplementary material for: Treatment of Periodontitis Affecting Human Primary Teeth—A Systematic Review
Source: Dent J (Basel). 2023 Jul 14;11(7):171. doi: 10.3390/dj11070171 (PMC10378644; doi:10.3390/dj11070171)
Supplement: Supplementary file 1 [file dentistry-11-00171-s001.zip › dentistry-2390679-supplementary.pdf]

Supplementary Material S1:

DETAILED SEARCH STRATEGY

PUBMED:

("aggressive periodontitis"[MeSH Terms] OR "aggressive periodontitis"[All Fields] OR "juvenile periodontitis"[All Fields] OR "pre-pubertal periodontitis"[All Fields] OR "rapidly progressing periodontitis"[All Fields] OR "early onset periodontitis"[All Fields]) AND ("tooth, deciduous"[MeSH Terms] OR "deciduous tooth"[All Fields] OR "primary teeth"[All Fields] OR "primary tooth"[All Fields] OR "deciduous teeth"[All Fields] OR "primary dentition"[All Fields] OR "deciduous dentition"[All Fields] OR "mixed dentition"[All Fields] OR "child"[MeSH Terms] OR "child"[All Fields] OR "children"[All Fields])

WEB OF SCIENCE:

1. ALL=(aggressive periodontitis OR juvenile periodontitis OR pre-pubertal periodontitis OR pre pubertal periodontitis OR rapidly progressing periodontitis OR early onset periodontitis)

2. ALL=(deciduous tooth OR primary teeth OR primary tooth OR deciduous teeth OR primary dentition OR deciduous dentition OR mixed dentition OR child OR children)

Final search: 1 AND 2

EMBASE:

1. 'aggressive periodontitis'/exp OR 'aggressive periodontitis' OR 'aggressive periodontitis':ti,ab,kw,de OR 'juvenile periodontitis':ti,ab,kw,de OR 'pre pubertal periodontitis':ti,ab,kw,de OR 'pre-pubertal periodontitis':ti,ab,kw,de OR 'rapidly progressing periodontitis':ti,ab,kw,de OR 'early onset periodontitis':ti,ab,kw,de
2. 'deciduous tooth'/exp OR 'deciduous tooth' OR 'deciduous tooth':ti,ab,kw,de OR 'primary teeth':ti,ab,kw,de OR 'primary tooth':ti,ab,kw,de OR 'deciduous teeth':ti,ab,kw,de OR 'primary dentition':ti,ab,kw,de OR 'deciduous dentition':ti,ab,kw,de OR 'mixed dentition'/exp OR 'mixed dentition' OR 'mixed dentition':ti,ab,kw,de OR 'child'/exp OR 'child' OR 'child':ti,ab,kw,de OR 'children':ti,ab,kw,de

Final Search: #1 AND #2

EBSCOHOST:

S1: (MH "Aggressive Periodontitis+")

S2: aggressive periodontitis OR juvenile periodontitis OR pre pubertal periodontitis OR pre-pubertal periodontitis OR rapidly progressing periodontitis OR early onset periodontitis

S3: (MH "Tooth, Deciduous+") OR (MH "Dentition, Primary+")

S4: (MH "Child+")

S5: deciduous tooth OR primary teeth OR primary tooth OR deciduous teeth OR primary dentition OR deciduous dentition OR mixed dentition OR child OR children

Final search: (S1 OR S2) AND (S3 OR S4 OR S5)

EXCLUDED ARTICLES

S. No	AUTHOR NAME	YEAR	REASON FOR EXCLUSION
1	Abbott [48]	1989	Unable to obtain full text of paper
2	Allin et al. [49]	2016	Outcomes for primary teeth not reported§
3	Barber et al. [50]	2012	Treatment outcomes not reported
4	Beliveau et al. [51]	2012	Permanent teeth
5	Bimstein et al. [25]	1997	Same patient population as in another paper
6	Bimstein [52]	2004	Review article
7	Bimstein et al. [12]	2004	Patient with immune deficiency
8	Bodur et al. [53]	2001	Permanent teeth
9	Branco-de-Almeida et al. [34]	2021	Outcomes for primary teeth not reported§
10	Budtz Jorgensen et al. [54]	1977	Unable to obtain full text of paper
11	Buduneli et al. [55]	2001	Treatment outcomes not reported
12	Burgess et al. [56]	2017	Outcomes for primary teeth not reported§
13	Butler [57]	1969	Permanent teeth
14	Celenligil et al. [58]	1987	Permanent teeth
15	Christersson et al. [59]	1985	Permanent teeth
16	Christersson et al. [60]	1993	Review article
17	Cogen et al. [61]	1984	Treatment outcomes not reported
18	Compton et al. [62]	1983	Unable to obtain full text of paper
19	Debevc and Silver [63]	2011	Review article
20	Dibart et al. [64]	1998	Treatment outcomes not reported
21	Dopico et al. [65]	2016	Permanent teeth
22	Dosumu et al. [66]	2015	Permanent teeth
23	Emingil et al. [67]	2001	Permanent teeth
24	Epstein [68]	1995	Permanent teeth
25	Fretwell et al. [69]	1981	Treatment outcomes not reported
26	Goepferd [70]	1981	Treatment outcomes not reported
27	Gonçalves et al. [71]	2013	Outcomes for primary teeth not reported§
28	Haring [72]	1999	Treatment outcomes not reported
29	Hempton et al. [73]	1997	Review article
30	Hoffman [74]	1983	Permanent teeth
31	Kalash et al. [75]	2015	Outcomes for primary teeth not reported§
32	Katz et al. [76]	1989	Treatment outcomes not reported
33	Kornman and Robertson [77]	1985	Permanent teeth
34	Kunihira et al. [78]	1985	Permanent teeth
35	Linden et al. [79]	1994	Patient did not follow through with specified treatment
36	Liyange et al. [80]	2016	Immune deficiency
37	Lopez [81]	1992	Permanent teeth

38	Mandell et al. [82]	1986	Follow-up clinical outcomes not reported
39	Mandell et al. [83]	1986	Permanent teeth
40	McIntyre [84]	1989	Review article
41	McLain et al. [85]	1983	Permanent teeth
42	Mengel et al. [86]	2002	Permanent teeth
43	Michel et al. [87]	1993	Outcomes of interest not reported
44	Mishkin et al. [88]	1986	Treatment outcomes not reported
45	Modin et al. [89]	2017	Permanent teeth
46	Mombelli et al. [90]	2013	Permanent teeth
47	Mombelli et al. [91]	2015	Permanent teeth
48	Monteiro et al. [92]	2020	Patients did not have aggressive periodontitis
49	Morimoto et al. [93]	2019	Immune deficiency
50	Muppa et al. [94]	2016	Treatment outcomes not reported
51	Myers et al. [95]	1989	Treatment outcomes not reported
52	Nowzari et al. [96]	2001	No details of treatment
53	Ogunsalu et al. [97]	2011	No details of treatment
54	Olgun-Erdemir et al. [98]	2010	Treatment outcomes not reported
55	Page et al. [99]	1985	Permanent teeth
56	Palmer et al. [100]	1996	Permanent teeth
57	Piergallini et al. [101]	2014	Language other than English
58	Prud'homme et al. [102]	2018	Treatment outcomes not reported
59	Qi et al. [103]	2021	Outcomes of interest not reported
60	Ram and Bimstein [104]	1994	Treatment outcomes not reported
61	Ramich et al. [105]	2018	Permanent teeth
62	Reis et al. [106]	2021	Permanent teeth
63	Rosenthal [107]	1951	Outcomes of interest not reported
64	Shaddox et al. [108]	2013	Outcomes for primary teeth not reported[§]
65	Shapira et al. [109]	1994	Permanent teeth
66	Sharma and Whatling [42]	2011	Treatment outcomes not reported
67	Sood et al. [110]	1988	Treatment outcomes not reported
68	Spektor et al. [111]	1985	No treatment discussed
69	Steiger et al. [112]	2008	Outcomes of interest not reported
70	Suzuki et al. [113]	1985	Permanent teeth
71	Tavakoli et al. [114]	2022	Outcomes for primary teeth not reported[§]
72	Tinoco et al. [115]	1998	Permanent teeth
73	Vandana and Redy [116]	2003	Treatment outcomes not reported
74	Velsko et al. [36]	2020	Outcomes for primary teeth not reported[§]
75	Watanabe [117]	1991	Permanent teeth
76	Yalcin et al. [118]	2001	Permanent teeth

Articles that were excluded after full-text screening, along with the reason for exclusion.

§ - Authors were contacted to request additional data from the study, to determine possible inclusion.

PRISMA CHECKLIST

Section and Topic	Item #	Checklist item	Location where item is reported
**TITLE**			
Title	1	Identify the report as a systematic review.	1
**ABSTRACT**			
Abstract	2	See the PRISMA 2020 for Abstracts checklist.	1
**INTRODUCTION**			
Rationale	3	Describe the rationale for the review in the context of existing knowledge.	2
Objectives	4	Provide an explicit statement of the objective(s) or question(s) the review addresses.	2
**METHODS**			
Eligibility criteria	5	Specify the inclusion and exclusion criteria for the review and how studies were grouped for the syntheses.	2,3
Information sources	6	Specify all databases, registers, websites, organisations, reference lists and other sources searched or consulted to identify studies. Specify the date when each source was last searched or consulted.	3
Search strategy	7	Present the full search strategies for all databases, registers and websites, including any filters and limits used.	Supplement 1
Selection process	8	Specify the methods used to decide whether a study met the inclusion criteria of the review, including how many reviewers screened each record and each report retrieved, whether they worked independently, and if applicable, details of automation tools used in the process.	3
Data collection process	9	Specify the methods used to collect data from reports, including how many reviewers collected data from each report, whether they worked independently, any processes for obtaining or confirming data from study investigators, and if applicable, details of automation tools used in the process.	3,4
Data items	10a	List and define all outcomes for which data were sought. Specify whether all results that were compatible with each outcome domain in each study were sought (e.g. for all measures, time points, analyses), and if not, the methods used to decide which results to collect.	3,4
	10b	List and define all other variables for which data were sought (e.g. participant and intervention characteristics, funding sources). Describe any	3,4

Section and Topic	Item #	Checklist item	Location where item is reported
		assumptions made about any missing or unclear information.	
Study risk of bias assessment	11	Specify the methods used to assess risk of bias in the included studies, including details of the tool(s) used, how many reviewers assessed each study and whether they worked independently, and if applicable, details of automation tools used in the process.	4
Effect measures	12	Specify for each outcome the effect measure(s) (e.g. risk ratio, mean difference) used in the synthesis or presentation of results.	4
Synthesis methods	13a	Describe the processes used to decide which studies were eligible for each synthesis (e.g. tabulating the study intervention characteristics and comparing against the planned groups for each synthesis (item #5)).	4
	13b	Describe any methods required to prepare the data for presentation or synthesis, such as handling of missing summary statistics, or data conversions.	4
	13c	Describe any methods used to tabulate or visually display results of individual studies and syntheses.	4
	13d	Describe any methods used to synthesize results and provide a rationale for the choice(s). If meta-analysis was performed, describe the model(s), method(s) to identify the presence and extent of statistical heterogeneity, and software package(s) used.	4
	13e	Describe any methods used to explore possible causes of heterogeneity among study results (e.g. subgroup analysis, meta-regression).	N/A
	13f	Describe any sensitivity analyses conducted to assess robustness of the synthesized results.	N/A
Reporting bias assessment	14	Describe any methods used to assess risk of bias due to missing results in a synthesis (arising from reporting biases).	N/A
Certainty assessment	15	Describe any methods used to assess certainty (or confidence) in the body of evidence for an outcome.	N/A
**RESULTS**			
Study selection	16a	Describe the results of the search and selection process, from the number of records identified in the search to the number of studies included in the review, ideally using a flow diagram.	4,5, Figure1
	16b	Cite studies that might appear to meet the inclusion criteria, but which were excluded, and explain why they were excluded.	Supplement 1
Study characteristics	17	Cite each included study and present its characteristics.	6-10
**Section and Topic**	**Item #**	**Checklist item**	**Location where item is reported**
Risk of bias in studies	18	Present assessments of risk of bias for each included study.	10-12
Results of individual studies	19	For all outcomes, present, for each study: (a) summary statistics for each group (where appropriate) and (b) an effect estimate and its precision (e.g. confidence/credible interval), ideally using structured tables or plots.	6-10
Results of syntheses	20a	For each synthesis, briefly summarise the characteristics and risk of bias among contributing studies.	10-12
	20b	Present results of all statistical syntheses conducted. If meta-analysis was done, present for each the summary estimate and its precision (e.g. confidence/credible interval) and measures of statistical heterogeneity. If comparing groups, describe the direction of the effect.	N/A
	20c	Present results of all investigations of possible causes of heterogeneity among study results.	N/A
	20d	Present results of all sensitivity analyses conducted to assess the robustness of the synthesized results.	N/A
Reporting biases	21	Present assessments of risk of bias due to missing results (arising from reporting biases) for each synthesis assessed.	N/A
Certainty of evidence	22	Present assessments of certainty (or confidence) in the body of evidence for each outcome assessed.	N/A
**DISCUSSION**			
Discussion	23a	Provide a general interpretation of the results in the context of other evidence.	12,13
	23b	Discuss any limitations of the evidence included in the review.	13
	23c	Discuss any limitations of the review processes used.	13
	23d	Discuss implications of the results for practice, policy, and future research.	13,14
**OTHER INFORMATION**			
Registration and protocol	24a	Provide registration information for the review, including register name and registration number, or state that the review was not registered.	2
	24b	Indicate where the review protocol can be accessed, or state that a protocol was not prepared.	2
	24c	Describe and explain any amendments to information provided at registration or in the protocol.	N/A
Support	25	Describe sources of financial or non-financial support for the review, and the role of the funders or sponsors in the review.	14

Section and Topic	Item #	Checklist item	Location where item is reported
Competing interests	26	Declare any competing interests of review authors.	14
Availability of data, code and other materials	27	Report which of the following are publicly available and where they can be found: template data collection forms; data extracted from included studies; data used for all analyses; analytic code; any other materials used in the review.	N/A

REFERENCES:

12. Bimstein, E.; McIlwain, M.; Katz, J.; Jerrell, G.; Primosch, R. Aggressive Periodontitis of the Primary Dentition Associated with Idiopathic Immune Deficiency: Case Report and Treatment Considerations. *J. Clin. Pediatr. Dent.* 2004, *29*, 27–31.
25. Bimstein, E.; Sela, M.N.; Shapira, L. Clinical and Microbial Considerations for the Treatment of an Extended Kindred with Seven Cases of Prepubertal Periodontitis: A 2-Year Follow-Up. *Pediatr. Dent.* **1997**, *19*, 396–403.
34. Branco-de-Almeida, L.S.; Cruz-Almeida, Y.; Gonzalez-Marrero, Y.; Kudsi, R.; de Oliveira, I.C.V.; Dolia, B.; Huang, H.; Aukhil, I.; Harrison, P.; Shaddox, L.M. Treatment of Localized Aggressive Periodontitis Alters Local Host Immunoinflammatory Profiles: A Long-Term Evaluation. *J. Clin. Periodontol.* **2021**, *48*, 237–248. https://doi.org/10.1111/jcpe.13404.
36. Velsko, I.M.; Harrison, P.; Chalmers, N.; Barb, J.; Huang, H.; Aukhil, I.; Shaddox, L. Grade C Molar-Incisor Pattern Periodontitis Subgingival Microbial Profile before and after Treatment. J. Oral Microbiol. 2020, 12, 1814674. https://doi.org/10.1080/20002297.2020.1814674.
42. Sharma, G.; Whatling, R. Case Report: Premature Exfoliation of Primary Teeth in a 4-Year-Old Child, a Diagnostic Dilemma. Eur. Arch. Paediatr. Dent. 2011, 12, 312–317. https://doi.org/10.1007/BF03262830.
48. Abbott, B. Periodontal disease in children and adolescents. *J. Macomb. Dent. Soc.* **1989**, *26*, 29.
49. Allin, N.; Cruz-Almeida, Y.; Velsko, I.; Vovk, A.; Hovemcamp, N.; Harrison, P.; Huang, H.; Aukhil, I.; Wallet, S.; Shaddox, L. Inflammatory Response Influences Treatment of Localized Aggressive Periodontitis.. *J. Dent. Res.* **2016**, *95*, 635–641,. https://doi.org/10.1177/0022034516631973.
50. Barber, S.; Day, P.; Judge, M.; Toole, E.O.; Fayle, S. Variant Carvajal syndrome with additional dental anomalies. *Int. J. Paediatr. Dent.* **2012**, *22*, 390–396. https://doi.org/10.1111/j.1365-263x.2012.01230.x.
51. Beliveau, D.; Magnusson, I.; Bidwell, J.A.; Zapert, E.F.; Aukhil, I.; Wallet, S.M.; Shaddox, L.M. Benefits of early systemic antibiotics in localized aggressive periodontitis: A retrospective study. *J. Clin. Periodontol.* **2012**, *39*, 1075–1081. https://doi.org/10.1111/jcpe.12001.
52. Bimstein, E. Periodontal Health and Disease in Children and Adolescents. *Pediatr. Clin. North Am.* **1991**, *38*, 1183–1207. https://doi.org/10.1016/s0031-3955(16)38194-9.
53. Bodur, A.; Bodur, H.; Bal, B.; Balos, K. Generalized aggressive periodontitis in a prepubertal patient: A case report. *Quin-Tessence Int.* **2001**, *32*, 303–308.
54. Budtz Jorgensen, E.; Ellegaard, J.; Ellegaard, B. CMI in juvenile periodontitis and the effect of levamisole treatment. *Allergol. Immunopathol.* **1977**, *5*, 314–315.
55. Buduneli, N.; Baylas, H.; Aksu, G.; Kütükçüler, N. Prepubertal periodontitis associated with chronic granulomatous disease. *J. Clin. Periodontol.* **2001**, *28*, 589–593. https://doi.org/10.1034/j.1600-051x.2001.028006589.x.

56. Burgess, D.K.; Huang, H.; Harrison, P.; Kompotiati, T.; Aukhil, I.; Shaddox, L.M. Non-Surgical Therapy Reduces Presence of JP2 Clone in Localized Aggressive Periodontitis. *J. Periodontol.* **2017**, *88*, 1263–1270. https://doi.org/10.1902/jop.2017.170285.
57. Butler, J.H. A Familial Pattern of Juvenile Periodontitis (Periodontosis). *J. Periodontol.* **1969**, *40*, 115–118. https://doi.org/10.1902/jop.1969.40.2.115.
58. Celenligil, H.; Kansu, E.; Eratalay, K.; Yavuzyilmaz, E. Prepubertal periodontitis. A case report with an analysis of lymphocyte populations. *J. Clin. Periodontol.* **1987**, *14*, 85–88. https://doi.org/10.1111/j.1600-051x.1987.tb00947.x.
59. Christersson, L.A.; Slots, J.; Rosling, B.G.; Genco, R.J. Microbiological and clinical effects of surgical treatment of localized juvenile periodontitis. *J. Clin. Periodontol.* **1985**, *12*, 465–476. https://doi.org/10.1111/j.1600-051x.1985.tb01382.x.
60. Christersson, L.A. Actinobacillus actinomycetemcomitans and localized juvenile periodontitis. Clinical, microbiologic and histologic studies. *Swed. Dent. J. Suppl.* **1993**, *90*, 1–46.
61. Caufield, P.W.; Stanley, D.H.; Donaldson, D.K. Periodontal disease in healthy children: Two clinical reports. *Pediatr. Dent.* **1984**, *6*, 41–45.
62. Compton, D.W.; Claiborne, W.J.; Hutchens, L.H. Combined periodontal, orthodontic and fixed prosthetic treatment of juvenile periodontitis: A case report.. *Int. J. Periodontics Restor. Dent.* **1983**, *3*, 20–33.
63. Debevc, T.M.; Silver, J.G. Periodontal diseases affecting children and young adults. *J. Can. Dent. Assoc.* **1996**, *62*, 650–652.
64. Dibart, S.; Chapple, I.L.; Skobe, Z.; Shusterman, S.; Nedleman, H.L. Microbiological findings in prepubertal periodontitis. A case report. *J. Periodontol.* **1998**, *69*, 1172–1175. https://doi.org/10.1902/jop.1998.69.10.1172.
65. Dopico, J.; Nibali, L.; Donos, N. Disease progression in aggressive periodontitis patients. A Retrospective Study. *J. Clin. Periodontol.* **2016**, *43*, 531–537. https://doi.org/10.1111/jcpe.12533.
66. Dosumu, E.B.; Bankole, O.O.; Dosumu, O.O. Simultaneous occurrence of periodontal and skin abscesses in a Nigerian girl: Case report. *Afr. J. Biomed. Res.* **2015**, *18*, 249–255.
67. Emingil, G.; Darcan, .; Keskinoğlu, A.; Kütükçüler, N.; Atilla, G. Localized aggressive periodontitis in a patient with type 1 diabetes mellitus: A case report. *J. Periodontol.* **2001**, *72*, 1265–1270. https://doi.org/10.1902/jop.2000.72.9.1265.
68. Epstein, S.R. Localized prepubertal periodontitis--nonsurgical treatment of an adolescent patient: A case report. *Pract. Periodontics Aesthet. Dent.* **1995**, *7*, 55–59.
69. Fretwell, L.D.; Leinbach, T.E.; Wiley, D.O. Juvenile periodontitis: Report of cases. *J. Am. Dent. Assoc.* **1982**, *105*, 1022–1025. https://doi.org/10.14219/jada.archive.1982.0376.
70. Goepferd, S.J. Advanced alveolar bone loss in the primary dentition: A case report. *J. Periodontol.* **1981**, *52*, 753–757. https://doi.org/10.1902/jop.1981.52.12.753.
71. Gonçalves, P.F.; Huang, H.; McAninley, S.; Alfant, B.; Harrison, P.; Aukhil, I.; Walker, C.; Shaddox, L.M. Periodontal treatment reduces matrix metalloproteinase levels in localized aggressive periodontitis. *J. Periodontol.* **2013**, *84*, 1801–1808. https://doi.org/10.1902/jop.2013.130002.
72. Haring, J.I. Case study. Early-onset periodontitis. *RDH* **1999**, *19*, 11–102.
73. Hempton, T.J.; Wilkins, E.; Lancaster, D. Localized juvenile periodontitis. *RDH* **1997**, *17*, 30–32.
74. Hoffman, I.D. Familial occurrence of juvenile periodontitis with varied treatment of one of the siblings with five-year follow-up: Case reports. *J. Periodontol.* **1983**, *54*, 44–49. https://doi.org/10.1902/jop.1983.54.1.44.
75. Kalash, D.; Vovk, A.; Huang, H.; Aukhil, I.; Wallet, S.M.; Shaddox, L.M. Influence of Periodontal Therapy on Systemic Lipopolysaccharides in Children with Localized Aggressive Periodontitis. *Pediatr. Dent.* **2015**, *37*, 35–40.

76. Katz, J.; Ben-Yehuda, A.; Machtei, E.E.; Danon, Y.L.; Metzker, A. Tumoral calcinosis associated with early onset periodontitis. *J. Clin. Periodontol.* **1989**, *16*, 643–646. https://doi.org/10.1111/j.1600-051x.1989.tb01033.x.
77. Kornman, K.S.; Robertson, P.B. Clinical and microbiological evaluation of therapy for juvenile periodontitis. *J. Periodontol.* **1985**, *56*, 443–446. https://doi.org/10.1902/jop.1985.56.8.443.
78. Kunihira, D.M.; Caine, F.A.; Palcanis, K.G.; Best, A.M.; Ranney, R.R. A clinical trial of phenoxymethyl penicillin for adjunctive treatment of juvenile periodontitis. *J. Periodontol.* **1985**, *56*, 352–358. https://doi.org/10.1902/jop.1985.56.6.352.
79. Linden, G.; Fleming, P.; Coulter, W.; Lynn, G. Localized prepubertal periodontitis in a 5-year-old child: Investigations and clinical observations over a 3-year period. *Int. J. Paediatr. Dent.* **2009**, *4*, 47–53. https://doi.org/10.1111/j.1365-263x.1994.tb00101.x.
80. Liyange, S.; Edgar, D.; Shields, M.D.; Linden, G.J. Gingival Inflammation and Aggressive Periodontitis in a Child with a Specific Antibody Deficiency. *Dent. Updat.* **2016**, *43*, 130–136. https://doi.org/10.12968/denu.2016.43.2.130.
81. López, N.J. Clinical, Laboratory, and Immunological studies of a family with a high prevalence of generalized prepubertal and juvenile periodontitis. *J. Periodontol.* **1992**, *63*, 457–468. https://doi.org/10.1902/jop.1992.63.5.457.
82. Mandell, R.L.; Siegal, M.D.; Umland, E. Localized juvenile periodontitis of the primary dentition. *ASDC J. Dent. Child* **1986**, *53*, 193–196.
83. Mandell, R.L.; Tripodi, L.S.; Savitt, E.; Goodson, J.M.; Socransky, S.S. The effect of treatment on *Actinobacillus actinomycetemcomitans* in localized juvenile periodontitis. *J. Periodontol.* **1986**, *57*, 94–99. https://doi.org/10.1902/jop.1986.57.2.94.
84. McIntyre, T.R. Treatment of juvenile periodontitis. *Ont. Dent.* **1989**, *66*, 16–17.
85. McLain, J.B.; Proffit, W.R.; Davenport, R.H. Adjunctive orthodontic therapy in the treatment of juvenile periodontitis: Report of a case and review of the literature. *Am. J. Orthod.* **1983**, *83*, 290–298. https://doi.org/10.1016/0002-9416(83)90224-5.
86. Mengel, R.; Lehmann, K.M.; Metke, W.; Wolf, J.; Flores-de-Jacoby, L. A telescopic crown concept for the restoration of partially edentulous patients with aggressive generalized periodontitis: Two case reports. *Int. J. Periodontics Restor. Dent.* **2002**, *22*, 129–137.
87. Michel, J.F.; Michel, M.G.; Nadan, J.; Nowzari, H. The street children of Manila are affected by early-in-life periodontal infection: Description of a treatment modality: Sea salt. *Refuat Hapeh Vehashinayim (1993)* **2013**, *30*, 6–13.
88. Mishkin, D.J.; Grant, N.C.; Bergeron, R.A.; Young, W.L. Prepubertal periodontitis: A recently defined clinical entity. *Pediatr. Dent.* **1986**, *8*, 235–238.
89. Modin, C.; Abadji, D.; Adler, L.; Jansson, L. Treatment compliance in patients with aggressive periodontitis—A retrospective case-control study. *Acta Odontol. Scand.* **2016**, *75*, 94–99. https://doi.org/10.1080/00016357.2016.1259497.
90. Mombelli, A.; Cionca, N.; Almaghlouth, A.; Décaillet, F.; Courvoisier, D.S.; Giannopoulou, C. Are There Specific Benefits of Amoxicillin Plus Metronidazole in*Aggregatibacter actinomycetemcomitans*-Associated Periodontitis? Double-Masked, Randomized Clinical Trial of Efficacy and Safety. *J. Periodontol.* **2013**, *84*, 715–724. https://doi.org/10.1902/jop.2012.120281.
91. Mombelli, A.; Almaghlouth, A.; Cionca, N.; Courvoisier, D.S.; Giannopoulou, C. Differential Benefits of Amoxicillin–Metronidazole in Different Phases of Periodontal Therapy in a Randomized Controlled Crossover Clinical Trial. *J. Periodontol.* **2015**, *86*, 367–375. https://doi.org/10.1902/jop.2014.140478.

92. Monteiro, M.F.; Tonelli, H.; Reis, A.A.; Casati, M.Z.; Silvério, K.G.; Junior, F.H.N.; Sallum, E.A.; Casarin, R.C.V. Triclosan toothpaste as an adjunct therapy to plaque control in children from periodontitis families: A crossover clinical trial. *Clin. Oral Investig.* **2020**, *24*, 1421–1430. https://doi.org/10.1007/s00784-019-03121-6.
93. Morimoto, S.; Hirano, K.; Tabata, K.; Asaumi, H.; Morikawa, Y.; Matsumi, Y.; Naka, S.; Matsumoto-Nakano, M. Case of autoimmune neutropenia with severe marginal periodontitis. *Pediatr. Dent. J.* **2019**, *29*, 138–145. https://doi.org/10.1016/j.pdj.2019.06.002.
94. Muppa, R.; Nallanchakrava, S.; Chinta, M.; Manthena, R.T. Nonsyndromic localized aggressive periodontitis of primary dentition: A rare case report. *Contemp. Clin. Dent.* **2016**, *7*, 262–264. https://doi.org/10.4103/0976-237x.183062.
95. Myers, D.R.; O'Dell, N.L.; Clark, J.W.; Cross, R.L. Localized prepubertal periodontitis: Literature review and report of case. *ASDC J. Dent. Child* **1989**, *56*, 107–111.
96. Nowzari, H.; Jorgensen, M.G.; Ta, T.T.; Contreras, A.; Slots, J. Aggressive periodontitis associated with Fanconi's anemia. A case report. *J. Periodontol.* **2001**, *72*, 1601–1606. https://doi.org/10.1902/jop.2001.72.11.1601.
97. Ogunsalu, C.; Daisley, H.; Akpaka, P.E. Prevalence and antimicrobial susceptibility pattern of pathogens isolated from patients with juvenile periodontitis in Jamaica: A prospective multi-centre study of 15 cases over a 15-year period. *West. Indian Med. J.* **2011**, *60*, 235–239.
98. Olgun-Erdemir, E.; Yildirim, M.S.; Karşiyaka, M. Generalized aggressive periodontitis in a child with 92, XXYY / 46,XY mosaicism: Report of a second case. *Turk. J. Pediatr.* **2010**, *52*, 94–96.
99. Page, R.C.; Vandesteen, G.E.; Ebersole, J.L.; Williams, B.L.; Dixon, I.L.; Altman, L.C. Clinical and laboratory studies of a family with a high prevalence of juvenile periodontitis. *J. Periodontol.* **1985**, *56*, 602–610. https://doi.org/10.1902/jop.1985.56.10.602.
100. Palmer, R.; Watts, T.L.P.; Wilson, R. A double-blind trial of tetracycline in the management of early onset periodontitis. *J. Clin. Periodontol.* **1996**, *23*, 670–674. https://doi.org/10.1111/j.1600-051x.1996.tb00592.x.
101. Piergallini, G.; Malerba, A.; Mazzucchelli, L.; Strohmenger, L. Generalized aggressive periodontitis in prepubertal age: Description and comparison of two cases. *Pediatr. Medica Chir.* **2014**, *36,* 95. https://doi.org/10.4081/pmc.2014.95.
102. Roy, E.; Soueidan, A.; Fouassier, M. Multidisciplinary Clinical Management of a Localized Aggressive Periodontitis diagnosed in a Child with Glanzmann\'s Thrombasthenia. *Int. J. Clin. Pediatr. Dent.* **2018**, *11*, 344–348. https://doi.org/10.5005/jp-journals-10005-1536.
103. Qi, G.; Yu, K.; Feng, Y.; Zhang, Y.; Shao, Q.; Yu, M.; Wang, Y.; Ren, L.; Zhu, D.; Yang, G.; et al. 1α,25-dihydroxyvitamin D3 promotes early osteogenic differentiation of PDLSCs and a 12-year follow-up case of early-onset vitamin D deficiency periodontitis. *J. Steroid Biochem. Mol. Biol.* **2021**, *208*, 105805. https://doi.org/10.1016/j.jsbmb.2020.105805.
104. Ram, D.; Bimstein, E. Subgingival bacteria in a case of prepubertal periodontitis, before and one year after extractions of the affected primary teeth. *J. Clin. Pediatr. Dent.* **1994**, *19*, 45–47.
105. Ramich, T.; Asendorf, A.; Nickles, K.; Oremek, G.M.; Schubert, R.; Nibali, L.; Wohlfeil, M.; Eickholz, P. Inflammatory serum markers up to 5 years after comprehensive periodontal therapy of aggressive and chronic periodontitis. *Clin. Oral Investig.* **2018**, *22*, 3079–3089. https://doi.org/10.1007/s00784-018-2398-x.
106. Reis, A.A.; Paz, H.E.D.S.; Monteiro, M.D.F.; Casati, M.Z.; Steiner-Oliveira, C.; Pascon, F.M.; Casarin, R.C.V. Early Manifestation of Periodontal Disease in Children and Its Association with Familial Aggregation. *J. Dent. Child.* **2021**, *88*, 140–143.
107. Rosenthal, S.L. Case Report: Periodontosis in a Child Resulting in Exfoliation of the Teeth. *J. Periodontol.* **1951**, *22*, 101–104. https://doi.org/10.1902/jop.1951.22.2.101.

108. Shaddox, L.; Gonçalves, P.; Vovk, A.; Allin, N.; Huang, H.; Hou, W.; Aukhil, I.; Wallet, S. LPS-induced inflammatory response after therapy of aggressive periodontitis. *J. Dent. Res.* **2013**, *92*, 702–708. https://doi.org/10.1177/0022034513495242.
109. Shapira, L.; Smidt, A.; Van Dyke, T.E.; Barak, V.; Soskolne, A.W.; Brautbar, C.; Sela, M.N.; Bimstein, E. Sequential manifestation of different forms of early-onset periodontitis. A case report. *J. Periodontol.* **1994**, *65*, 631–635. https://doi.org/10.1902/jop.1994.65.6.631.
110. Sood, P.B.; Sood, M.; Mescarenhas, A.K. Prepubertal periodontitis--a case report. *J. Indian Soc. Pedod. Prev. Dent.* **1988**, *6*, 45–47.
111. Spektor, M.D.; Vandesteen, G.E.; Page, R.C. Clinical studies of one family manifesting rapidly progressive, juvenile and prepubertal periodontitis. *J. Periodontol.* **1985**, *56*, 93–101. https://doi.org/10.1902/jop.1985.56.2.93.
112. Steiger, J.; Zeevi, A.; Bhola, M. Aggressive periodontitis: A case report. *J. Mich. Dent. Assoc.* **2008**, *90*, 40–46.
113. Suzuki, J.B.; Risom, L.; Falkler, W.A.; Collison, C.; Bowers, G. Effect of periodontal therapy on spontaneous lymphocyte response and neutrophil chemotaxis in localized and generalized juvenile periodontitis patients*. *J. Clin. Periodontol.* **1985**, *12*, 124–134. https://doi.org/10.1111/j.1600-051x.1985.tb01371.x.
114. Tavakoli, T.T.; Gholami, F.; Huang, H.; Gonçalves, P.F.; Villasante-Tezanos, A.; Aukhil, I.; Oliveira, R.C.G.; Hovencamp, N.; Wallet, S.; Ioannidou, E.; et al. Gender differences in immunological response of African-American juveniles with Grade C molar incisor pattern periodontitis. *J. Periodontol.* **2021**, *93*, 392–402. https://doi.org/10.1002/jper.21-0143.
115. Tinoco, E.M.; Beldi, M.I.; Campedelli, F.; Lana, M.; Loureiro, C.A.; Bellini, H.T.; Rams, T.E.; Tinoco, N.M.; Gjermo, P.; Preus, H.R. Clinical and microbiological effects of adjunctive antibiotics in treatment of localized juvenile periodontitis. A controlled clinical trial. *J. Periodontol.* **1998**, *69*, 1355–1363. https://doi.org/10.1902/jop.1998.69.12.1355.
116. Vandana, K.L.; Redy, B.V.R. Prepubertal periodontitis: A report of 2 cases. *J. Dent. Child (Chic)* **2003**, *70*, 82–85.
117. Watanabe, K. Generalized juvenile periodontitis in a thirteen-year-old child. *ASDC J. Dent. Child* **1991**, *58*, 390–395.
118. Yalçın, S.; Yalçın, F.; Günay, Y.; Bellaz, B.; Önal, Ş.; Firatli, E. Treatment of aggressive periodontitis by osseointegrated dental implants. A case report. *J. Periodontol.* **2001**, *72*, 411–416. https://doi.org/10.1902/jop.2001.72.3.411.
